# Editorial: Ion and Water Transport in Cell Death

**DOI:** 10.3389/fcell.2021.757033

**Published:** 2021-09-09

**Authors:** Markus Ritter, Alexander A. Mongin, Giovanna Valenti, Yasunobu Okada

**Affiliations:** ^1^Center for Physiology, Pathophysiology and Biophysics, Institute for Physiology and Pathophysiology, Paracelsus Medical University, Salzburg, Austria; ^2^Kathmandu University, Dhulikhel, Nepal; ^3^Ludwig Boltzmann Institute for Arthritis and Rehabilitation, Salzburg, Austria; ^4^Gastein Research Institute, Paracelsus Medical University, Salzburg, Austria; ^5^Department of Neuroscience and Experimental Therapeutics, Albany Medical College, Albany, NY, United States; ^6^Department of Biosciences, Biotechnologies and Biopharmaceutics, University of Bari Aldo Moro, Bari, Italy; ^7^Division of Cell Signaling, National Institute for Physiological Sciences (NIPS), Okazaki, Japan; ^8^Department of Physiology, School of Medicine, Aichi Medical University, Nagakute, Japan; ^9^Department of Physiology, Kyoto Prefectural University of Medicine, Kyoto, Japan

**Keywords:** cell death, ion transport, water transport, apoptosis, necrosis, cell volume regulation, cell hydration

The flow of water across cellular membranes determines the dynamics of cellular hydration and volume, both of which govern a plentitude of fundamental functions, including cell death (CD) (Lang et al., 1993, 1998, 2007; Haussinger, 1996; Hoffmann et al., 2009; Okada et al., 2021; Okada et al.). The aforementioned reviews suggest that changes in the directionality of water transport involve a variety of mechanisms; they can be osmotically obligated in their nature and coupled to the movement of ions and organic osmolytes, driven by hydrostatic pressure, or determined by “ingestion” and “excretion” during endo/exocytotic processes.

The significance of water transport and cell volume in CD has been recognized for a long time. Injurious cell swelling, which was initially described as oncosis (from Greek Óγκ*o*ς, i.e. tumor/swelling), represents a hallmark of the unregulated form of CD—necrosis (von Recklinghausen, 1910; Majno and Joris, 1995; Weerasinghe and Buja, 2012). Similar increases in cell volume are prominent in several other related modes of CD, such as secondary necrosis (aponecrosis), pyroptosis, and ferroptosis (Formigli et al., 2000; Zong and Thompson, 2006; Silva, 2010; D'Arcy, 2019; Okada et al., 2019; Nirmala and Lopus, 2020; Riegman et al., 2020). While necrosis was initially considered to be a result of uncontrolled water accumulation, recent studies suggest that this form of CD starts with tightly controlled normotonic cell swelling, termed necrotic volume increase (NVI) (Barros et al., 2001; Okada et al., 2001, 2021; Orlov and Hamet, 2004; Lang and Hoffmann, 2013a,b; Orlov et al., 2013a,b; Bortner and Cidlowski, 2014; Model, 2014; Bortner and Cidlowski). In contrast, the highly regulated mode of CD, apoptosis, is generally associated with cell volume decrease (Majno and Joris, 1995; Lang et al., 1998; Maeno et al., 2000; Hoffmann et al., 2009). Apoptosis is initiated by the early and precisely regulated normotonic cell shrinkage, termed apoptotic volume decrease (AVD), which is driven by activation of distinct ion channels and transporters (Maeno et al., 2000; Okada et al., 2001). Additionally, the emerging research indicates that the precise regulation of ion and water transport across organellar membranes is also indispensable for normal cell function, and its disturbances may cause CD and disease (Maltese and Overmeyer, 2014; Li et al., 2020; Saric and Freeman, 2020; Chadwick et al., 2021; Bouteau et al.; Ritter et al.; Urbani et al.). Apart from the two major types of CD, apoptosis and necrosis, numerous additional (sub)forms of CD have been identified. These include aponecrosis, oncosis, necroptosis, parthanatos, anoikis, entotic CD, NETotic CD, immunogenic CD, lysosome-dependent CD, ferroptosis, oxeiptosis, sarmoptosis, autosis, autolysis, paraptosis, pyroptosis, alkaliptosis, phagoptosis, eryptosis, chondroptosis, autophagic CD, mitoptosis, methuosis, and the mitotic catastrophe-driven CD (Galluzzi et al., 2018). Although all these forms of CD involve very diverse and distinct mechanisms (Green and Llambi, 2015; Galluzzi et al., 2018; Nirmala and Lopus, 2020), their successful execution relies on tightly regulated transport of ions, organic solutes and water across the plasma membrane and/or organelle membranes (Lang et al., 1998; Okada and Maeno, 2001; Chen et al., 2008; Hoffmann et al., 2009; Orlov et al., 2013c; Maltese and Overmeyer, 2014; Mongin, 2016; Okada et al., 2019, 2021; Okada et al.). Understanding these mechanisms is crucial for our comprehension of the basic principles of normal and abnormal biological processes.

The objective of this Research Topic was to collect state-of-the-art Reviews and cutting-edge original research articles exploring ions and water transport in cell death. This timely subject has attracted significant enthusiasm of the scientific community. The initial Call for Contributions resulted in 23 accepted manuscripts covering various aspects of the pivotal roles of ion and water transport in cell death. The collection encompasses four Original Research papers (Centeio et al.; Kittl et al.; Wei et al.; Yurinskaya et al.), two Brief Research Reports (Rana and Model; Matsuura et al.), 11 Reviews (Bachmann et al.; Bortner and Cidlowski; Bose et al.; Chen et al.; Dias et al.; Foller and Lang; Kim et al.; Lefranc; Ritter et al.; Okada et al.; Shimizu et al.), four Mini Reviews (Kolbrink et al.; Urbani et al.; Amiri et al.; Shiozaki et al.), one Hypotheses article (Shen et al.), and one Opinion article (Bouteau et al.) These publications are contributed by 98 authors. While in the production, the Research Topic was met with high interest within the scientific community; it accumulates the steadily increasing number of views and downloads from all parts of the world (https://www.frontiersin.org/research-topics/13260/ion-and-water-transport-in-cell-death#impact).

The articles in this Research Topic cover a large variety of types of CD, including apoptosis (Bachmann et al.; Bortner and Cidlowski; Okada et al.; Rana and Model; Shimizu et al.; Urbani et al.; Yurinskaya et al.; Shiozaki et al.; Lefranc), necrosis (Bouteau et al.; Kittl et al.; Okada et al.; Lefranc), aponecrosis (Wei et al.), necroptosis and pyroptosis (Kolbrink et al.; Okada et al.), ferroptosis (Chen et al.; Okada et al.; Shen et al.; Lefranc), paraptosis (Kim et al.), eryptosis (Dias et al.; Foller and Lang), methuosis (Ritter et al.) as well as plant vacuolar CD (Bouteau et al.). From the standpoint of the mechanisms of CD-inducing processes, the Topic contributors discuss the functional significance of numerous specific anion channels, cation channels, and ion transporters (Bachmann et al.; Bortner and Cidlowski; Bouteau et al.; Kim et al.; Kittl et al.; Kolbrink et al.; Okada et al.; Rana and Model; Shen et al.; Shimizu et al.; Urbani et al.; Wei et al.; Yurinskaya et al.; Amiri et al.; Lefranc; Ritter et al.; Shiozaki et al.). To facilitate reading of this collection, we provide cross-references to the related ion transport mechanisms in Supplementary Tables 1–3 (referring to anion channels, cation channels, and transporters, respectively). Additionally, this collection discusses the important roles in CD for the water channels, aquaporins (AQPs) (Bortner and Cidlowski; Shiozaki et al.), and mechanistic contributions for some organic signaling molecules (such as ATP, glutamate and glutathione) which are released *via* anion channels (Okada et al.; Matsuura et al.).

It is also important to place each CD type in the context of the pathogenesis of different human diseases. The contributors discuss the role of apoptosis in ischemia/reperfusion (I/R) injury, including excitotoxicity (Okada et al.), chronic neurodegenerative disorders, including Alzheimer's disease (Bachmann et al.), cancer (Shiozaki et al.), and chemoresistance of cancer (Bachmann et al.; Okada et al.; Shimizu et al.); necrosis in I/R injury including lactacidotoxicity (Okada et al.), osteoarthritis (Kittl et al.), and cancer (Lefranc); aponecrosis in I/R injury (Wei et al.); necroptosis and pyroptosis in I/R injury, neurodegeneration, cancer, skin inflammation, and crystallopathies (Kolbrink et al.); ferroptosis in I/R injury (Chen et al.; Shen et al.), neurodegeneration (Chen et al.; Rana and Model), cancer (Chen et al.), and acute CNS injury (Shen et al.); paraptosis in I/R injury and chemoresistance of cancer (Kim et al.); as well as eryptosis in anemia and chronic kidney disease (CKD) (Dias et al.; Foller and Lang). There is a hope in the field that further elucidation of molecular mechanisms of ion and water transport underlining these CD processes is likely to provide accurate targets for therapy of CD-associated diseases and for the treatment of chemo-resistant cancer.

Collectively, the contributions from the Research Topic emphasize the progress in the field of physiological/pathophysiological mechanisms of cell death and their roles in the pathogenesis of various diseases.

## Author Contributions

All authors contributed equally to the editorial work of this Research Topic and to this Editorial and approved it for publication.

## Funding

This work in the laboratories of the Research Topic editors was supported by National Institutes of Health (grants R01 NS061953, R01 NS11943 to AAM), the Japan Society for the Promotion of Science (Grant-in-Aid for Scientific Research #17K19517 to YO), POR Puglia Innonetwork (regional project H6GG787 to GV), and Agenzia Spaziale Italiana (GV).

## Conflict of Interest

The authors declare that the research was conducted in the absence of any commercial or financial relationships that could be construed as a potential conflict of interest.

## Publisher's Note

All claims expressed in this article are solely those of the authors and do not necessarily represent those of their affiliated organizations, or those of the publisher, the editors and the reviewers. Any product that may be evaluated in this article, or claim that may be made by its manufacturer, is not guaranteed or endorsed by the publisher.
