# Supplementary material for: Editorial: Ion and Water Transport in Cell Death
Source: Front Cell Dev Biol. 2021 Sep 9;9:757033. doi: 10.3389/fcell.2021.757033 (PMC8458750; doi:10.3389/fcell.2021.757033)
Supplement: Supplementary file 1 [file Table_1.pdf]

TABLE 1. Anion channels reported to be implicated in the CD induction/protection in this Research Topic.

Anion channels	Specified cell death types	References
VSOR/VRAC*[1]	apoptosis	(Bortner and Cidlowski, 2020;Okada et al., 2020;Shiozaki et al., 2021)
	necrosis (lactacidotoxicity)	(Okada et al., 2020)
	necrosis (acidotoxicity)	(Kittl et al., 2020)
	methuosis	(Ritter et al., 2021)
	pyroptosis and necroptosis	(Kolbrink et al., 2020)
ASOR/PAC*[2]	necrosis (acidotoxicity)	(Kittl et al., 2020)
	methuosis	(Ritter et al., 2021)
TMEM16A/ANO1*[3]	apoptosis	(Shiozaki et al., 2021)
	methuosis	(Ritter et al., 2021)
Maxi-Cl*[4]	necrosis (excitotoxicity)	(Okada et al., 2020)
	eryptosis	(Foller and Lang, 2020)
CFTR*[5]	apoptosis	(Shiozaki et al., 2021)
	methuosis	(Ritter et al., 2021)
ClC-2	apoptosis	(Shiozaki et al., 2021)
Intracellular CLIC*[6]	apoptosis	(Shiozaki et al., 2021)
	paraptosis	(Kim et al., 2020)
Mitochondrial VDAC*[7]	paraptosis	(Kim et al., 2020)
$GABA_A$ receptor	necrosis (excitotoxicity)	(Okada et al., 2020)
Plant Cl^- channel	necrosis and vacuolar cell death	(Bouteau et al., 2020)

*[1] VSOR: volume-sensitive outwardly rectifying anion channel; also called VRAC: volume-regulated anion channel; *[2] ASOR: acid-sensitive outwardly rectifying anion channel; also called PAC: proton-activated anion channel; *[3] TMEM16A: transmembrane protein 16A; also called ANO1: anoctamin-1
*[4] Maxi-Cl: large-conductance maxi-anion channel; *[5] CFTR: cystic fibrosis transmembrane regulator; that is, cAMP-activated anion channel; *[6] CLIC: chloride intracellular ion channel; *[7] VDAC: Voltage-dependent anion channel; this is a mitochondrial porin.
