# Supplementary material for: Editorial: Ion and Water Transport in Cell Death
Source: Front Cell Dev Biol. 2021 Sep 9;9:757033. doi: 10.3389/fcell.2021.757033 (PMC8458750; doi:10.3389/fcell.2021.757033)
Supplement: Supplementary file 2 [file Table_2.pdf]

TABLE 2. Cation channels reported to be implicated in the CD induction/protection in this Research Topic.

**Cation channels**		**Specified cell death types**	**References**
Non-selective cation channels	TRPs (transient receptor potential channels)	apoptosis	(Kolbrink et al., 2020;Lefranc, 2021)
		aponecrosis*	(Wei et al., 2020)
		methuosis	(Ritter et al., 2021)
		eryptosis	(Dias et al., 2020;Foller and Lang, 2020)
	TPCs (two-pore channels)	methuosis	(Ritter et al., 2021)
	iGluR (ionotropic glutamate receptor)	apoptosis	(Bortner and Cidlowski, 2020)
		necrosis (excitotoxicity)	(Okada et al., 2020)
		eryptosis	(Dias et al., 2020)
	PIEZO1 (mechanosensitive ion channel)	eryptosis	(Dias et al., 2020)
Na^+ channel	Na_v (voltage-gated Na^+ channel)	methuosis	(Ritter et al., 2021)
K^+ channel	K_v (voltage-gated K^+ channel)	apoptosis	(Bachmann et al., 2020;Bortner and Cidlowski, 2020;Urbani et al., 2020;Shiozaki et al., 2021)
		methuosis	(Ritter et al., 2021)
	K_{Ca} (Ca^{2+}-activated K^+ channel)	apoptosis	(Okada et al., 2020;Shiozaki et al., 2021)
		methuosis	(Ritter et al., 2021)
		eryptosis	(Dias et al., 2020;Foller and Lang, 2020)
	K_{2P} (two-pore-domain K^+ channel)	apoptosis	(Bortner and Cidlowski, 2020;Shiozaki et al., 2021)
	mK_{ATP} (mitochondrial ATP-sensitive K^+ channel)	apoptosis	(Bortner and Cidlowski, 2020)
	plant K^+ channel	necrosis or vacuolar cell death	(Bouteau et al., 2020)

Ca^{2+} channel	Ca_v (voltage-gated Ca^{2+} channel)	apoptosis	(Shiozaki et al., 2021)
		eryptosis	(Foller and Lang, 2020)
	IP_3R (inositol 1,4,5-trisphosphate receptor)	paraptosis	(Kim et al., 2020)
	RyR (ryanodine receptor)	paraptosis	(Kim et al., 2020)

*aponecrosis: also called the secondary necrosis which is generated after abortion of apoptotic processes on the way due to an incomplete execution of the apoptosis program often caused by ATP deficiency (Formigli et al., 2000;Silva, 2010;Okada et al., 2019), thus exhibiting both some apoptotic biochemical events such as caspase-3 activation and necrotic events such as NVI, as observed in this article (Wei et al., 2020).
