# Supplementary material for: Editorial: Ion and Water Transport in Cell Death
Source: Front Cell Dev Biol. 2021 Sep 9;9:757033. doi: 10.3389/fcell.2021.757033 (PMC8458750; doi:10.3389/fcell.2021.757033)
Supplement: Supplementary file 3 [file Table_3.pdf]

TABLE 3. Inorganic and organic ion transporters reported to be implicated in the CD induction/protection in this Research Topic.

**Ion transporters**		**Specified cell death types**	**References**
Uniporters	OCT (organic cation transporter)	apoptosis	(Shimizu et al., 2020)
	CTR (copper transporter 1)	apoptosis	(Shimizu et al., 2020)
	SLC39/40A1/25A37/25A20*	ferroptosis	(Dias et al., 2020)
	MCU (mitochondrial Ca^{2+} uniporter	apoptosis	(Urbani et al., 2020)
		paraptosis	(Kim et al., 2020)
	DMT1 (endosomal divalent metal transporter 1)	ferroptosis	(Shen et al., 2020)
Symporters	NKCC (Na^{+}-K^{+}-$2Cl^{-}$ cotransporter)	apoptosis	(Yurinskaya et al., 2020)
	NCC (Na^{+}-Cl^{-} cotransporter)	apoptosis	(Yurinskaya et al., 2020)
	KCC (K^{+}-Cl^{-} cotransporter)	apoptosis	(Yurinskaya et al., 2020)
	MCT (monocarboxylate transporter)	necrosis (lactacidotoxicity)	(Okada et al., 2020)
Antiporters	NHE (Na^{+}/H^{+} exchanger)	necrosis (lactacidotoxicity)	(Okada et al., 2020)
		methuosis	(Ritter et al., 2021)
	NCX (Na^{+}/Ca^{2+} exchanger)	eryptosis	(Dias et al., 2020)
	AE (anion exchanger or Cl^{-}/HCO_3^{-} exchanger)	apoptosis	(Shiozaki et al., 2021)
		necrosis (lactacidotoxicity)	(Okada et al., 2020)
		eryptosis	(Foller and Lang, 2020)
	ClC (intracellular Cl^{-}/H^{+} exchanger)	methuosis	(Ritter et al., 2021)
	mNCX (mitochondrial NCX)	apoptosis	(Urbani et al., 2020)
		paraptosis	(Kim et al., 2020)

	mHCX (mitochondrial H^+/Ca^{2+} exchanger)	paraptosis	(Kim et al., 2020)
Pumps	Na^+-K^+ pump (Na^+-K^+ ATPase)	apoptosis	(Rana and Model, 2020;Yurinskaya et al., 2020)
		necrosis	(Okada et al., 2020)
		methuosis	(Ritter et al., 2021)
		eryptosis	(Foller and Lang, 2020)
	Ca^{2+} pump (PMCA: plasmamembrane Ca^{2+} ATPase)	eryptosis	(Dias et al., 2020;Foller and Lang, 2020)
	vATPase (vesicular proton pump)	apoptosis	(Shiozaki et al., 2021)
	MRP2 (multidrug resistance-associated protein 2)	apoptosis	(Shimizu et al., 2020)
	ATP7A/7B (copper-transporting ATPase 7A or 7B)	apoptosis	(Shimizu et al., 2020)

*SLC39/40A1/25A37/25A20: solute carrier family 39/40A1/25A37/25A20 members which are mitochondrial iron transporters.
